# King Edward's Fund Grants

**Published:** 1920-05-08

**Authors:** 


### KING EDWARD'S FUND GRANTS.

On a later page (137) we publish a report of the proceedings at the annual meeting of the President and General Council of King Edward's Hospital Fund for London, to receive the accounts and the report for the year 1919. As in the earlier statement issued by the honorary secretaries and given in our issue of March 27, the essential facts of the situation are put forward in a particularly accurate and convincing form, accompanied by just that degree of optimism with which those holding the voluntary hospitals' interests truly at heart should face the great difficulties ahead. Lord Somerleyton's opening tribute to Sir Henry Burdett, the news of whose death had been received on the eve of the meeting, was received with that appreciation given only to the memory of a great-hearted and able man; and one, indeed, who had never missed a meeting of the Council since the foundation of the fund twenty-three years ago. In this, as in so many spheres, his work has been monumental, and the memory of a life of unfaltering endeavour has provided a stimulus and an example which has been but rarely equalled in the records of organised charity.

Among the various announcements made, we notice that the amount of money distributed has been increased from £200,000 to £230,000, with a view to assisting the hospitals to meet the cost of such renewals and repairs as were postponed under the stress of the period of hostilities. It was estimated that £50,000 would therefore have to be withdrawn from the reserves created by exceptional legacies and donations received during the war, but actually this call proved to be about £4,000 less than was anticipated at the time; although this is, in itself, satisfactory, we see that coincident with the urgency of repairs, there has been an excessively rapid increase in the needs of hospitals for the ordinary purposes of maintenance. In 1918 this expenditure was 41 per cent. in excess of that experienced in 1913. Last year it was even greater, and the recently issued estimate puts the total for the London hospitals in 1919 at about £2,100,000. Against this, including their grants from the three Central Funds, the hospitals' income amounted to £1,900,000, leaving no more than a 10 per cent. deficit in the year's work—namely £200,000. It is, however, interesting to recall that at the time of the Fund's inauguration the corresponding annual deficit was about £70,000, which also represented 10 per cent. of the approximate expenditure at the particular period.

The fact, therefore, that in spite of all their recent troubles the London hospitals, after twenty-three years, still find themselves with the same percentage difficulties—no better, but happily no worse—sounds a more hopeful note; but we must encourage no feeling of false security for 1920, when the present steady rise of prices seems likely to some extent to persist, and when, though the deficit expressed as a percentage, is the same, the actual total is so very much greater. The situation in the institution arises automatically with other great financial effects of the war, but outside its walls the money and the people who hold it are still with us. True, there has been no little change in its distribution among the people of Great Britain and the power to give greatly has in many cases changed hands, but it is equally true that there are still some 95 per cent. of the population who at present contribute nothing to support of the hospitals. As was urged at the meeting, and as we have reiterated again and again in these pages, this lump sum deficit, large though it is, can be overcome by great and resolute efforts. But these must lead to a driving home of the facts to the heart of every London citizen, not to the wealthy alone, not particularly to the new-rich. What is needed is the reliable flow of hundreds of thousands of small subscriptions.

